# Metacommunity Structure of Stream Insects across Three Hierarchical Spatial scales

**DOI:** 10.1002/ece3.6103

**Published:** 2020-03-03

**Authors:** Siwen He, Janne Soininen, Guiping Deng, Beixin Wang

**Affiliations:** ^1^ Department of Entomology Nanjing Agricultural University Nanjing China; ^2^ Department of Geosciences and Geography University of Helsinki Helsinki Finland; ^3^ Jiuzhaigou Administrative Bureau Jiuzhaigou County China

**Keywords:** China, dispersal limitation, dispersal routes, environmental filtering, mass effects, variation partitioning

## Abstract

A major challenge in community ecology is to understand the underlying factors driving metacommunity (i.e., a set of local communities connected through species dispersal) dynamics. However, little is known about the effects of varying spatial scale on the relative importance of environmental and spatial (i.e., dispersal related) factors in shaping metacommunities and on the relevance of different dispersal pathways. Using a hierarchy of insect metacommunities at three spatial scales (a small, within‐stream scale, intermediate, among‐stream scale, and large, among‐sub‐basin scale), we assessed whether the relative importance of environmental and spatial factors shaping metacommunity structure varies predictably across spatial scales, and tested how the importance of different dispersal routes vary across spatial scales. We also studied if different dispersal ability groups differ in the balance between environmental and spatial control. Variation partitioning showed that environmental factors relative to spatial factors were more important for community composition at the within‐stream scale. In contrast, spatial factors (i.e., eigenvectors from Moran's eigenvector maps) relative to environmental factors were more important at the among‐sub‐basin scale. These results indicate that environmental filtering is likely to be more important at the smallest scale with highest connectivity, while dispersal limitation seems to be more important at the largest scale with lowest connectivity. Community variation at the among‐stream and among‐sub‐basin scales were strongly explained by geographical and topographical distances, indicating that overland pathways might be the main dispersal route at the larger scales among more isolated sites. The relative effect of environmental and spatial factors on insect communities varied between low and high dispersal ability groups; this variation was inconsistent among three hierarchical scales. In sum, our study indicates that spatial scale, connectivity, and dispersal ability jointly shape stream metacommunities.

## Graphical abstract

We compared metacommunity structure of aquatic insects across three hierarchical spatial scales. We found that the relative role of environmental and spatial factors and of different dispersal routes on community structure varied across spatial scales. These results indicate that stream systems respond to assembly processes differently at a variety of spatial scale.

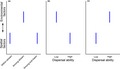

## 1 INTRODUCTION

Community ecology has moved toward focusing on the variability of regionally opened communities rather than understanding of the organization of locally closed communities (Leibold et al., 2004). Current views suggest that local communities are not only influenced by local‐scale environmental factors but also by spatial (i.e., dispersal related) factors operating at regional scales (Leibold & Chase, 2018). Metacommunity theory provides a useful framework to describe the underlying environmental and spatial factors influencing community composition in a set of local communities connected through species dispersal (Leibold et al., 2004). Recent studies suggest that metacommunities are often structured by a combination of environmental and spatial factors that vary in their relative importance (Heino et al., 2015; Sarremejane et al., 2017).

Typically, the balance between environmental and spatial control is affected by some fundamental factors related to spatial scale (Declerck, Coronel, Legendre, & Brendonck, 2011; Mykrä, Heino, & Muotka, 2007; Viana & Chase, 2019) or among‐site dispersal rates (Brown & Swan, 2010). At very small spatial scales or in well‐connected systems, there may occur excessive dispersal or mass effects (ME, local species composition being affected by high dispersal through source‐sink relations, Leibold & Chase, 2018), which allows species to persist also in environmentally unsuitable habitats due to the high level of dispersal from adjacent suitable habitats. This would contribute to the low influence of environmental control of local communities (Heino et al., 2015; Mouquet & Loreau, 2003). However, when spatial scale increases but remains moderate, different outcomes may emerge, and a metacommunity would display an increased match between environment and community composition. This is because (a) more environmental heterogeneity could be captured at larger spatial scales and dispersal would be weaker between more distant sites; (b) weaker dispersal does not lead to mass effects but allow species to track variation in environmental heterogeneity across sites. Finally, at very large spatial scales or in regions with major dispersal barriers, there may occur dispersal limitation, which prevents species from reaching their suitable habitats, leading also to a weak match between environment and biotic community (Horváth, Vad, & Ptacnik, 2016; Ng, Carr, & Cottenie, 2009).

Dispersal routes used by individuals may also affect metacommunity organization. An idealized model system for the study of dispersal routes using dispersal proxies is a stream network, where dispersal routes are diverse and complex (Brown & Swan, 2010; Göthe, Angeler, & Sandin, 2013; Tonkin et al., 2018). For example, some dispersal is restricted within the stream corridors (e.g., drifting insect larvae dispersal, Giller & Malmqvist, 1998) while some dispersal also occurs out‐of‐stream (e.g., adult flying insect dispersal, Petersen, Masters, Hildrew, & Ormerod, 2004). Recent studies suggest that the usefulness of analyzing different dispersal routes to indicate the strengths of different types of dispersal or community assembly in streams were inconsistent (Tonkin et al., 2018), and likely to depend on spatial scale. Previous studies found that network (watercourse) dispersal across well‐connected streams and rivers was more important than overland dispersal at relatively small spatial scale (up to 50 km, Brown & Swan, 2010; Padial et al., 2014; Rouquette et al., 2013). In contrast, Sarremejane et al. (2017) have shown that dispersal through overland (i.e., topographical and geographical routes) is more important than dispersal through watercourse within a stream network at large scale (up to 260 km). However, very few studies have investigated the relative importance of different dispersal routes on community assembly at multiple spatial scales.

**Figure 1:**
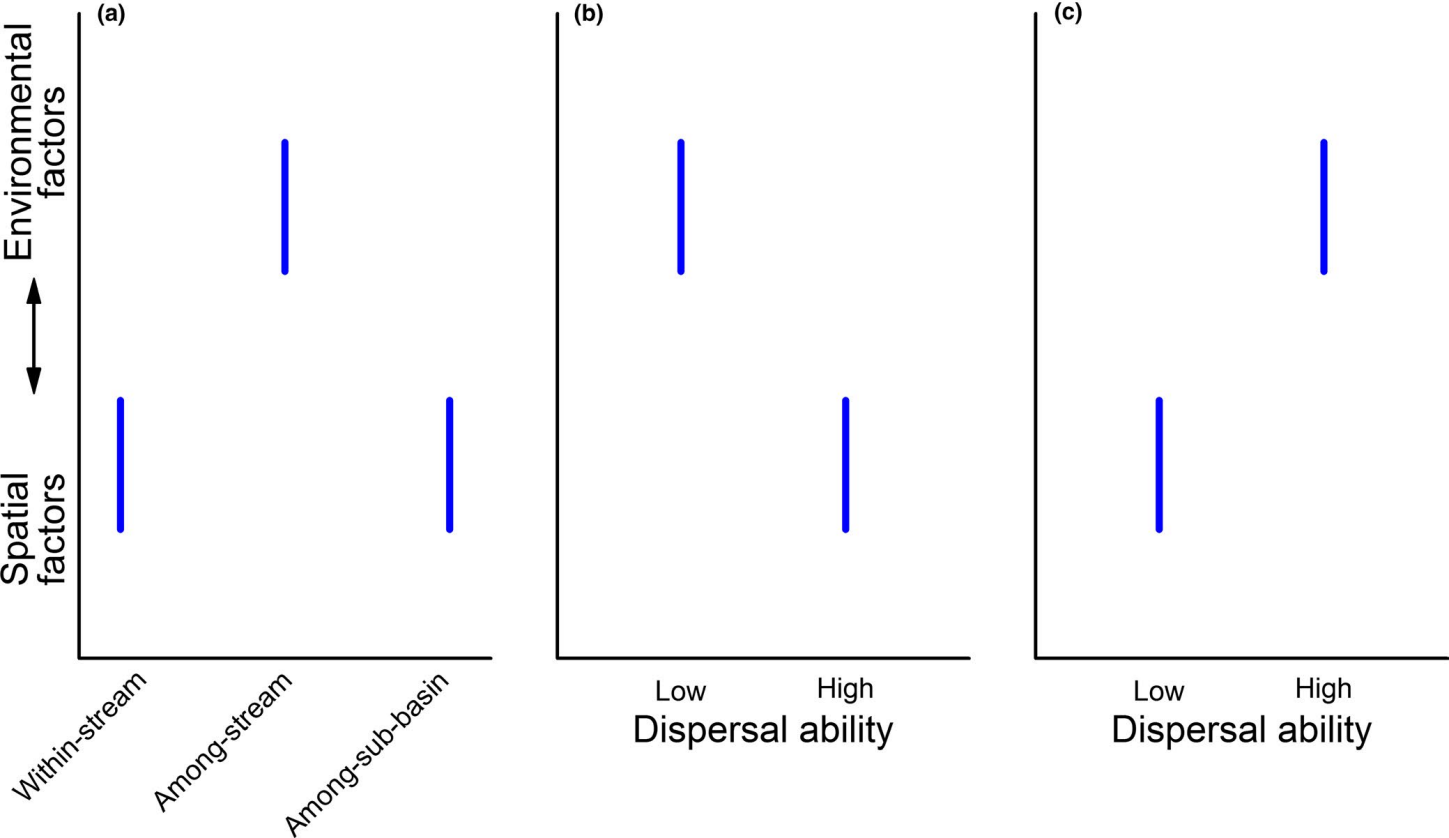
Expected relative effect of environmental and spatial factors on metacommunities across the three levels of spatial scales (i.e., within‐stream scale, among‐stream scale, and among‐sub‐basin scale) (a) and between different dispersal ability groups separately at the smallest scale (b) and the larger scales (c)

In this study, we investigated the structure of multiple stream insect metacommunities at three hierarchical scales (i.e., within‐stream scale, among‐stream scale, and among‐sub‐basin scale) to test the following hypotheses: (H1) Insects are expected to be more affected by spatial factors (i.e., eigenvectors from Moran's eigenvector maps) relative to environmental factors (e.g., pH and water depth) at the smallest, within‐stream scale due to ME and the largest, among‐sub‐basin scale due to dispersal limitation (Figure 1a) (Heino et al., 2015). However, at an intermediate, among‐stream scale, insects are more likely to be affected by environmental factors than spatial factors. (H2) Out‐of‐stream dispersal would be stronger at the larger scales, where organisms need to rely more on overland dispersal across streams or basins, whereas watercourse dispersal would be stronger at the smallest scale among well‐connected neighboring sites.

The relative roles of environmental and spatial drivers on community composition may also vary with species traits, especially dispersal ability (Astorga et al., 2012; Cottenie, 2005). Aquatic insects are key organisms in stream communities and comprise diverse species groups with different dispersal abilities. We thus also hypothesized that the relative roles of environmental and spatial factors structuring different dispersal ability groups vary among spatial scales: (H3a) at the within‐stream scale, insects with high dispersal ability are expected to be more affected by spatial factors relative to environmental factors (Figure 1b) because they are more likely to exhibit ME than those with low dispersal ability (Tonkin, Stoll, Jähnig, & Haase, 2016); while (H3b) at the among‐stream and among‐sub‐basin scales, insects with high dispersal ability are expected to be more affected by environmental factors relative to spatial factors (Figure 1c), because they track the environmental variation well (Grönroos et al., 2013) and are less dispersal limited than those with low dispersal ability. Here, we tested these hypotheses with stream invertebrate data collected in three drainage basins in China.

## 2 MATERIALS AND METHODS

### 2.1 Study area

**Figure 2:**
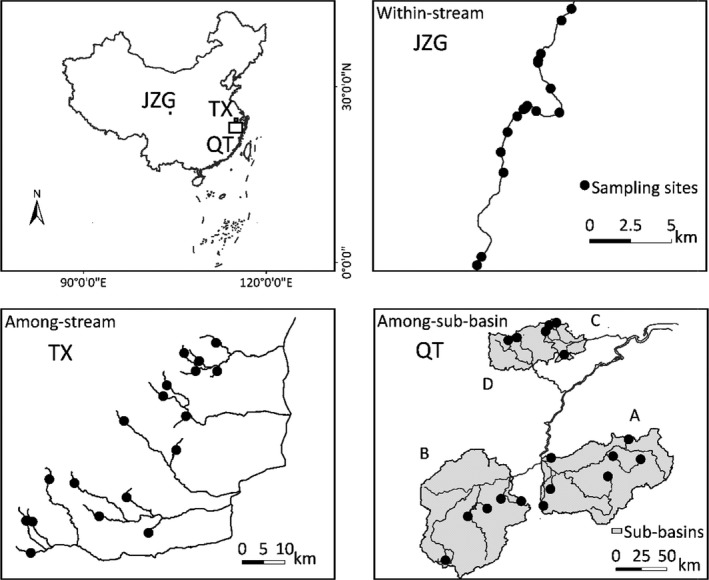
Map of the sample sites in three study regions in China: Jiuzhaigou Nature Reserve (JZG), Tiaoxi River Basin (TX), and Qiantang River Basin (QT). JZG represents the within‐stream scale, TX the among‐stream scale, and QT the among‐sub‐basin scale. A, B, C, and D represent four sub‐basins in the Qiantang River Basin

We used three datasets with total of 54 sites comprising stream insects sampled from different‐sized regions in China: Jiuzhaigou Nature Reserve (JZG; 643 km^2^), Tiaoxi River Basin (TX; 4,100 km^2^), and Qiantang River Basin (QT; 55,000 km^2^) (Figure 2). For the first dataset (hereafter referred to as the JZG dataset), we collected 18 sites within one stream (Wang et al., 2018). For the second dataset (TX dataset), we collected one site in each of 18 streams. The total number of sites sampled for the TX dataset thus equals 18. For the third dataset (QT dataset), we selected a number of streams in each of four sub‐basins (7 streams in sub‐basin A, 5 in sub‐basin B, 4 in sub‐basin C, and 2 in sub‐basin D, Figure 2) and in each of these streams we collected one site. In total, the QT dataset consists of 18 sampled sites from four sub‐basins. Therefore, three datasets differed in spatial scale and represented the different levels of the stream hierarchy (Frissell, Liss, Warren, & Hurley, 1986), but had the same number of sites. All sites are relatively undisturbed with >75% forested land use. We note here that not only the spatial scale varied among the datasets, but also connectivity as in the JZG dataset, all sites were directly connected while the others covered multiple streams or sub‐basins in which species need to rely on overland dispersal.

### 2.2 Biotic sampling

We used a 30 cm D‐frame net with 250 μm mesh size in JZG (Wang et al., 2018) and TX, and a 30 cm Surber net with 250 μm mesh size in QT (Wang et al., 2012) to sample insect assemblage. Samples (each sample with 30‐s kicking) were applied to various habitats (e.g., riffles, runs, pools, and dead woods) along a 100m reach or the wadeable areas. The number of samples was similar at each site in TX and in QT (Wang et al., 2012) but differed among sites (ranged from 5 to 10) in JZG (Wang et al., 2018). Samples referred to here as “sample units” and were combined into one composite sample at each site. In the laboratory, insect individuals were counted and identified to the lowest practical taxonomic level (usually genus).

### 2.3 Environmental variables

At each site, 10 environmental variables were measured (Table 1). We used a METTLER TOLEDO meter (model SG23, Mettler) to measure water temperature (WT), dissolved oxygen (DO), pH, total dissolved solids (TDS), and conductivity (Cond) in situ. We used a portable meter HI93752 (Hanna) to measure calcium (Ca^2+^) concentrations. Elevation was documented with a Garmin eTrex. Prior to the field measurements and biotic sampling, we collected one 500 ml water sample at each riffle and stored them in a portable refrigerator at <4°C. In the laboratory, we analyzed the samples for total nitrogen (TN) and total phosphorus (TP). Water depth (WD) was measured at each site based on five cross‐stream transects.

**Table 1:** Mean, minimum, maximum, and standard deviation (*SD*) of 10 environmental variables, species richness, and abundance at sample sites at the three levels of spatial scales (i.e., within‐stream scale, among‐stream scale, and among‐sub‐basin scale). Also, the watercourse, geographical and topographical distances between sites are shown

	Within‐stream			Among‐stream			Among‐sub‐basin		
	Mean	Min‐max	*SD*	Mean	Min‐max	*SD*	Mean	Min‐max	*SD*
Water temperature (℃)	10.70	6.90–14.00	2.00	23.60	17.80–27.90	3.20	16.10	12.40–22.50	2.90
pH	8.30	8.20–8.60	0.10	8.10	6.90–9.60	0.60	7.60	6.00–9.70	0.90
Dissolved oxygen (mg/L)	7.30	6.80–7.90	0.30	8.30	4.60–10.40	1.20	9.80	8.70–10.80	0.60
Total dissolved solids (mg/L)	233.30	203.00–252.00	16.00	67.80	30.70–110.30	20.70	24.70	9.00–55.00	11.80
Conductivity (μs/cm)	347.10	303.00–371.00	22.70	125.50	20.20–221.00	46.10	49.90	17.00–114.00	24.60
Calcium concentrations (mg/L)	75.50	56.70–133.70	17.90	64.80	1.00–141.00	41.50	38.90	2.00–90.00	29.40
Total nitrogen (mg/L)	0.28	0.10–0.54	0.14	1.23	0.56–2.47	0.71	0.98	0.20–2.03	0.52
Total phosphorus (mg/L)	0.01	0.00–0.02	0.01	0.09	0.00–0.49	0.13	0.02	0.00–0.05	0.02
Elevation (km)	2.44	2.19–2.91	0.20	0.15	0.03–0.52	0.14	0.34	0.14–0.70	0.13
Water depth (cm)	29.00	15.00–91.70	18.70	19.10	10.90–29.80	5.80	16.90	0.20–36.70	7.30
Local richness	22.00	7.00–31.00	8.00	34.00	12.00–50.00	10.00	43.00	23.00–68.00	13.00
Local abundance	701.00	12.00–1401.00	377.00	1,152.00	76.00–3280.00	765.00	1559.00	321.00–3296.00	936.00
Geographical distances (km)	4.90	0.04–16.40	3.80	26.20	1.30–61.90	15.30	112.60	6.60–250.60	60.80
Topographical distances (km)	5.20	0.07–17.80	4.10	52.40	3.20–135.80	34.40	200.50	20.10–471.50	109.90
Watercourse distances (km)	6.70	0.07–21.20	4.90	83.50	6.40–139.20	35.60	243.40	17.40–464.30	109.40

### 2.4 Distance metrics

We used three distance metrics, which described different potential pathways to disperse. These were as follows: (a) Geographical distances as the Euclidean straight‐line distances between sites in 2‐dimensional space, (b) topographical distances as the distance of sites across elevational barriers, and (c) watercourse distance as the distance of sites along stream channels were calculated using the Analysis/Proximity/Point distance tool, the 3D Analyst/Functional Surface/Add Surface Information tool and the Network Analyst/Make OD Cost Matrix and Add location tools, respectively, in ArcGIS 10.3 software.

### 2.5 Connectivity measures

We estimated the metacommunity connectivity for stream insects following Yeh et al. (2015) and measured as:

$$\text{Avg}\text{. Con}.=\frac{1}{n}\sum_{i=1}^{n}\text{Con}._{i}$$

$$\text{Con}._{i}=\frac{1}{m}\frac{1}{n-1}\sum_{\begin{array}{c} j=1\\ j\neq i \end{array}}^{n}\sum_{k=1}^{m}p_{\mathrm{jk}}\exp\;\left(-d_{\mathrm{ij}}\right)$$

Avg. Con. is the average of site connectivities, *d_ij_* is the distance between site *i* (focal site) and *j* (surrounding site), *p* indicates the presence or absence of *k*th taxa in the *j*th site, *n* is the total number of sites, and *m* is the total number of taxa in the site pair (i.e., site *i* and *j*). Analyses of metacommunity connectivity were conducted in R version 3.2.2 (R Core Team,^2016^).

### 2.6 Dispersal classifications

In our study, we used two different measures of dispersal ability following Poff et al. (2006): drifting propensity and female dispersal and assigned each insect taxon into two classes: Low and high dispersal ability (see Table S1 for details of classification). Because dispersal ability (drifting propensity and female dispersal) of different genera of the same family was generally similar (Poff et al., 2006), we assigned dispersal ability at family level. Families that were not included in Poff et al. (2006) were excluded from the analysis.

### 2.7 Data analysis

#### 2.7.1 Variation partitioning

We used classical and Moran Spectral Randomization (MSR, Wagner & Dray, 2015) based variation partitioning (VP, Borcard, Legendre, & Drapeau, 1992; Clappe, Dray, & Peres‐Neto, 2018) to tease apart the relative importance of environmental and spatial control in three datasets (hypothesis H1) and between different dispersal ability groups (H3). Prior to the VP analyses, the biological abundance data were Hellinger transformed (Legendre & Gallagher, 2001) and environmental variables were log (*X* + 1)‐transformed. We only used abundance data, as flying insect abundance data relative to presence‐absence data are expected to be more likely affected by ME (Bie et al., 2012), ME being potentially one of the main interests of our paper. We used the Moran's eigenvector maps (MEM, Dray, Legendre, & Peres‐Neto, 2006) framework to detect spatial structure of insect communities. We firstly used a data‐driven approach to select the best spatial weighting matrix (SWM) following a recent study (Silberberger, Renaud, Buhl‐Mortensen, Ellingsen, & Reiss, 2019). We then computed the MEMs based on the selected SWM (see Table S2 and S3 for details of selection). To facilitate the comparisons across different datasets, we ran forward selection and retained the first four positive MEM eigenvectors and the first four environmental variables in each dataset. With the retained variables, we partitioned the total amount of explained community variation into the following parts: variation uniquely explained by environmental factors ([E|S]), variation uniquely explained by spatial factors ([S|E]), spatially structured environmental variation ([E ∩ S]), and unexplained variation. We used the difference between [E|S] and [S|E] to distinguish the relative dominance of environmental and spatial control (Padial et al., 2014).

As the sizes of species pool were expected to affect the level of adjusted R^2^ in VP (Siqueira et al., 2012), we standardized the number of stream taxa in each dataset to *S* (integers). We formed a pooled species matrix combing all taxa in each dataset, and randomly resampled this matrix to form groups of *S* “taxa.” Therefore, the “*S*” used here is different to the “sample units” used in the biotic sampling. We chose *S* = 20 in all cases (except the case of JZG for high female dispersal, *S = *15) and conducted the standardizing exercise 500 times. In the case of all insect taxa group only, we also considered *S* = 30, 40, and 50 (above 50 taxa were not available).

#### 2.7.2 Linear mixed‐effect model

We applied the linear mixed‐effect model (LME) to assess the effect of different distances on community similarity of insects (H2). We calculated the dissimilarity index using Bray–Curtis coefficients based on abundance data (Legendre & Legendre, 2012). The fixed and random effect terms in the LME are the explanatory factors (i.e., geographical, topographical, and watercourse distances) and the dependency between the pairwise distances. We used minimum likelihood population effect (MLPE, Clarke, Rothery, & Raybould, 2002) to account for the nonindependence of the distance matrices. We assessed the amount of variation explained by fixed effects using the $R_{\beta }^{2}$ estimate derived from Kenward–Roger's estimate (Edwards, Muller, Wolfinger, Qaqish, & Schabenberger, 2008; Sarremejane et al., 2017).

#### 2.7.3 Environmental heterogeneity

We used an analysis of homogeneity of group dispersions (PERMDISP, Anderson, 2006) to test the differences in the degree of environmental heterogeneity (mean distances of sites to group centroid) among the three datasets. We used ANOVA *F*‐statistic to compare within‐group distances to each group centroid and tested the significance of the differences among groups with 1,000 permutations.

#### 2.7.4 Diversity partitioning

Understanding diversity patterns may be beneficial for distinguish ME, environmental filtering and dispersal limitation as environmentally filtered and dispersal‐assembled (i.e., either under ME or dispersal limitation) metacommunities may show different diversity patterns (Tonkin et al., 2016). We partitioned the total gamma diversity into local alpha (within sites) diversity and beta (among sites in each dataset) diversity (Lande, 1996) in each dataset and used the standardized effect size (SES) to examine if these partitions were greater than expected by chance through comparison with null matrices, using 999 permutations. We constrained the null matrix using “r2table” method, which fixes both row and column totals. Diversity partitioning analyses were performed only for entire data because we removed some families in the dispersal trait analysis due to the lack of dispersal ability information.

We conducted all statistical analyses in R version 3.2.2 using the packages “ade4” (Dray & Dufour, 2007) and “adespatial” (Dary et al., 2017) for MSR, “lme4” (Bates, Machler, Bolker, & Walker, 2015) for MLPE, and “pbkrtest” (Halekoh & Højsgaard, 2014) for the Kenward‐Roger's estimates, and “vegan” (Oksanen et al., 2016) for PERMDISP and diversity partitioning analyses.

## 3 RESULTS

### 3.1 Distance metrics and connectivity measures

For each type of distance, the range and mean values between sites were the lowest at the within‐stream scale (c.f. JZG dataset), intermediate at the among‐stream scale (TX dataset) and the highest at the among‐sub‐basin scale (QT dataset) (Table 1). At each spatial scale, geographical distances covered the shortest range and had lowest mean values compared with topographical and watercourse distances (Table 1). The metacommunity connectivity was the highest at the within‐stream scale, intermediate at the among‐stream scale, and lowest at the among‐sub‐basin scale (Table 2), regardless of the distance metric used. At each spatial scale, the metacommunity connectivity was the highest with geographical distance, lower with topographical, and watercourse distances (Table 2).

**Table 2:** Summary of determination coefficients (R^2^_β_) for the relationships between community dissimilarity and geographical (GEO), topographical (TOP), and watercourse (WAT) distances, and of metacommunity connectivities (Avg. Con.) for different taxa groups across the three hierarchical spatial scales

Taxa group	Spatial scale	$R_{\beta }^{2}$			Avg. Con. ╳ 10^–3^		
		GEO	TOP	WAT	GEO	TOP	WAT
All insect	Within‐stream	0.017	0.006	0.003	74.486	73.263	61.475
	Among‐stream	0.301	0.193	0.026	2.105	0.244	0.019
	Among‐sub‐basin	0.327	0.140	0.004	0.014	<0.001	<0.001
Low drifting propensity	Within‐stream	0.053	0.053	0.043	56.266	54.885	45.782
	Among‐stream	0.351	0.235	0.005	1.581	0.182	0.015
	Among‐sub‐basin	0.191	0.132	0.000	0.013	<0.001	<0.001
High drifting propensity	Within‐stream	0.019	0.007	0.004	88.735	87.633	73.697
	Among‐stream	0.283	0.189	0.023	2.452	0.284	0.023
	Among‐sub‐basin	0.305	0.113	0.000	0.015	<0.001	<0.001
Low female dispersal	Within‐stream	0.023	0.012	0.002	72.452	71.113	59.281
	Among‐stream	0.328	0.162	0.041	2.007	0.232	0.018
	Among‐sub‐basin	0.297	0.209	0.000	0.014	<0.001	<0.001
High female dispersal	Within‐stream	0.068	0.080	0.112	90.027	88.969	75.066
	Among‐stream	0.193	0.197	0.001	2.406	0.279	0.023
	Among‐sub‐basin	0.052	0.004	0.027	0.015	<0.001	<0.001

### 3.2 Environmental heterogeneity

Environmental heterogeneity differed among the three spatial scales based on PERMDISP analysis (*F*_2, 51_ = 6.09, *p* = .004). The within‐stream scale showed the lowest environmental variation (mean Euclidean distance to group centroid ± Standard Error: 1.20 ± 0.27) while it was higher at the among‐sub‐basin scale (2.02 ± 0.18) and especially the among‐stream scale (2.45 ± 0.30).

### 3.3 Comparisons between environmental and spatial control

**Figure 3:**
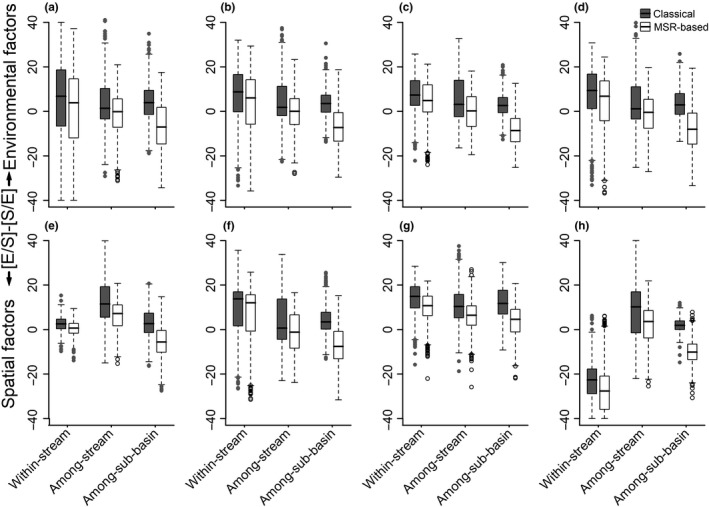
Boxplot of the difference between pure effect of environmental factors ([E|S]) and pure effect of spatial factors ([S|E]) from Classical and Moran Spectral Randomization (MSR) based variance partitioning for insects across the three levels of spatial scales. Analyses were performed separately for all insects randomized with 20 (a), 30 (b), 40 (c) and 50 (d) number of taxa, Low drifting propensity (e), High drifting propensity (f), Low female dispersal (g), and High female dispersal (h), respectively. Boxes represent interquartile range; central bar represents the median; dots are outliers (>1.5 × interquartile range)

We found that the difference between [E|S] and [S|E] varied strongly across three hierarchical spatial scales (Figure 3). In most cases (e.g., for all insect taxa), [E|S]‐[S|E] was lower at the among‐stream and among‐sub‐basin scales than at the within‐stream scale (Figure 3). We also found that [E|S]‐[S|E] peaked at the among‐stream scale, but only for low drifting propensity and high female dispersal (Figure 3e,h), partly agreeing with hypothesis H1. The results of classical VP analysis showed patterns in the [E|S]**‐**[S|E] across three spatial scales similar to the findings based on the MSR‐based analysis, and the values of the [E|S]**‐**[S|E] were consistently higher when the classical analysis was used, especially at the among‐sub‐basin scale.

At the within‐stream scale, [E|S]‐[S|E] was considerably lower for insects with higher female dispersal (Figure 3g,h), but higher for insects with higher drifting propensity (Figure 3e,f), partly supporting H3a. At the among‐stream and among‐sub‐basin scales, [E|S]‐[S|E] was generally lower for stronger dispersers, disagreeing with H3b.

### 3.4 Comparison between different distance metrics

The geographical and topographical distance had generally high explanatory power over community dissimilarity at the among‐stream and among‐sub‐basin scales (average values of R^2^_β_ for geographical and topographical distance were 0.23 and 0.12 at the among‐stream scale, and 0.29 and 0.20 at the among‐sub‐basin scale; Table 2). In contrast, the watercourse distance had generally low explanatory power over community dissimilarity in all cases (Table 2).

## 4 DISCUSSION

Dendritic systems, such as streams, are spatially structured in a hierarchical manner. Studies have largely ignored the hierarchical spatial structure of stream systems (but see Heino & Grönroos, 2013; Jyrkänkallio‐Mikkola, Heino, & Soininen, 2016), and we therefore do not fully understand the implications of such a structure. One implication for changing hierarchical spatial scale is that it typically alters the relative importance of environmental and spatial factors on metacommunity structure. Here, we present evidence that the relative strengths of underlying drivers behind metacommunities differ depending on hierarchical spatial scale in stream systems, and that they also differ for low versus high dispersal ability groups.

### 4.1 Variation of metacommunity dynamics across spatial scales

**Figure 4:**
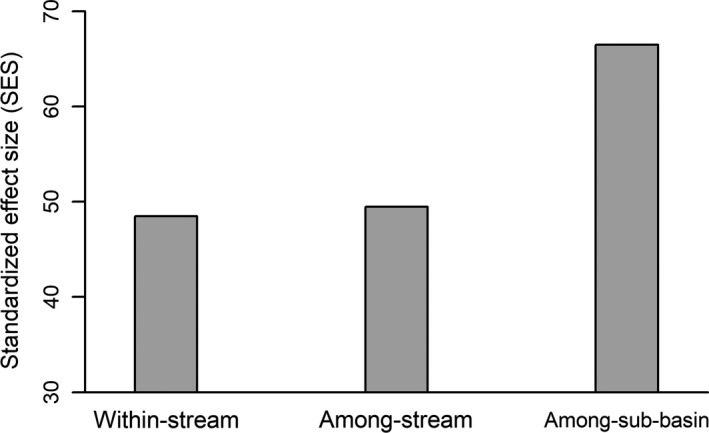
Standardized effect size (SES) for beta diversity of the diversity partitioning of insects at the three levels of spatial scales (i.e., within‐stream scale, among‐stream scale, and among‐sub‐basin scale). Due to the absolute values of SES for alpha and beta diversity are same, the SES only for beta diversity are showing. The observed values are significantly (*p* < .001) higher than the expected values at each spatial scale

Our results did not completely follow hypothesis H1, as the [E|S]‐[S|E] (i.e., the difference between environmental and spatial control) was the highest at the within‐stream scale and the lowest at the among‐sub‐basin scales. These findings indicate that environmental filtering was more important for community composition at the smallest scale, while dispersal limitation was more important at the largest scale. Similarly, Declerck et al. (2011) studied zooplankton metacommunities at three hierarchical spatial scales and found a stronger evidence of environmental control at the smallest spatial scale and a stronger evidence of spatial control (possibly dispersal limitation) at the largest spatial scale. Such a result may also arise here because species at the within‐stream scale are better able to track suitable habitats across the whole study extent due to efficient dispersal (Grönroos et al., 2013) and short distances among sites (i.e., high connectivity). In contrast, at the among‐sub‐basin scale, species may be less likely to disperse across sites to reach their suitable habitats due to dispersal limitation and long distances among sites (i.e., low connectivity), thus interfering with environmental control (Horváth et al., 2016; Ng et al., 2009). In addition, at the among‐sub‐basin scale, the effect of spatial factors was mainly modeled by broad‐scale MEM variables (e.g., MEM1 and MEM2, Table S2), further suggesting that dispersal limitation was the key driving force of spatial structure (De Bie et al., 2012). Such a suggestion was also supported by the diversity partitioning results. We found that the observed beta diversity among sites was significantly higher (*p* < .001, Figure 4) than expected by chance at each spatial scale. However, at the among‐sub‐basin scale, the difference between the observed and expected beta diversity was the largest (i.e., the standardized effect size was the highest), indicating that dispersal limitation was more important at the largest scale.

### 4.2 Comparison between different distance metrics

As hypothesized (H2), at the among‐stream and among‐sub‐basin scales with low connectivity, geographical and topographical distances were more related with community dissimilarity than watercourse distances. Similarly, previous studies have found evidence that overland distances were explaining better community dissimilarities than watercourse distances at large scales (Sarremejane et al., 2017) and in stream network subjected to hydrology isolation (Maloney & Munguia, 2011). The long watercourse distance between sites at the among‐stream and among‐sub‐basin scales might represent a natural or anthropogenic barrier for instream dispersers and constrain the dispersal of stream insects. Therefore, stream insects may be more laterally (geographically and topographically) dispersed across streams to search their suitable habitats (Cañedo‐Argüelles et al., 2015). However, at the within‐stream scale, each distance type did not relate well with community dissimilarity. Landeiro, Magnusson, Melo, Espirito‐Santo, and Bini (2011) similarly found that both overland and watercourse distances were equally poor descriptors of caddisfly community composition at small scale (total investigated area *c*. 100 km^2^). Collectively, our results suggest that out‐of‐stream dispersal was more important at the larger scales where sites are more isolated.

### 4.3 Comparison between low and high dispersal strength groups

At the within‐stream scale, we detected considerably lower [E|S]**‐**[S|E] for high female dispersal communities relative to low female dispersal communities, as was hypothesized (H3a). This result indicated that taxa with higher female dispersal were more dispersal assembled at the smallest scale. Moreover, the abundances of high female dispersal taxa varied clearly among adjacent sites at the within‐stream scale (Figure S1), possibly resulting in high downstream‐upstream movements among adjacent sites (Göthe et al., 2013). However, we did not find support for H3b at the among‐stream and among‐sub‐basin scales, where the [E|S]**‐**[S|E] was generally higher for lower dispersal taxa. These results suggested that low dispersal taxa were more environmentally filtered than high dispersal taxa at the larger scales. These findings may arise because even the high dispersal taxa were probably not able to reach all sites at the among‐stream and among‐sub‐basin scales due to excessive spatial extents or presence of major barriers (Tonkin et al., 2017). On the other hand, the low dispersal taxa would be more able to passively find suitable habitats at the larger scales or in isolated region because they benefit more from dispersal by wind (Göthe et al., 2013; Mouquet & Loreau, 2003) or animal vectors. In summary, the relative importance of environmental and spatial factors on community composition varied between low and high dispersal ability groups; and this variation was inconsistent among hierarchical spatial scales.

### 4.4 Comparison of two variation partitioning methods

Compared to the classical VP analysis, the MSR‐based VP procedure had considerably lower estimates of [E|S]‐[S|E] (i.e., higher estimates of [S|E] and lower estimates of [E|S]) at the among‐sub‐basin scale (Table S4, Figure 3), as Clappe et al. (2018) suggested. These results indicate that the classical [E ∩ S] was partly driven by spurious spatial autocorrelations at the among‐sub‐basin scale where the environment and species distributions were more spatially structured (Table S4). Therefore, if we had not applied the MSR‐based VP analysis into our dataset, we would have missed a spatial signal at the among‐sub‐basin scale.

### 4.5 Possible caveats

A potential caveat for our study may stem from inconsistent sampling surveys with three data set collected with different methods. As the stream width differed along the stream reach at the within‐stream scale, we sampled different number of samples among sites for perfect insect detection as possible. However, we found that the patterns of community similarity and results of VP analyses based on D‐frame net and Surber‐net data were highly similar at the within‐stream scale. Similarly, Brown et al. (2018) found no difference between different sample data for macroinvertebrates in Iceland and European Alps. We thus believe that our main conclusions of insect metacommunity assembly may have been only little affected by the influence of sample design.

## 5 CONCLUSIONS

By performing a comparative analysis of three hierarchical metacommunities in stream networks, we showed that the relative roles of environmental and spatial factors on community composition are likely to be scale‐dependent. At the within‐stream scale, environmental filtering may be stronger due to the smaller scale and higher connectivity compared with among‐stream and among‐sub‐basin scales. In contrast, at among‐sub‐basin scale, insect communities were perhaps more governed by dispersal limitation. We also suggested that insect dispersal at the among‐stream and among‐sub‐basin scales occurred mainly overland through the geographical and topographical routes. Finally, our analyses of dispersal traits indicate that the relative roles of environmental and spatial factors on stream metacommunities depend not only on spatial scale but also on dispersal ability. Overall, our results demonstrate complex metacommunity organization in hierarchical stream systems and suggest that spatial scale, connectivity, and dispersal ability jointly shape stream metacommunities.

## CONFLICT OF INTEREST

No conflict of interest to declare.

## AUTHOR CONTRIBUTIONS

S. H. and B. W. conceived the ideas, S. H. and J. S. analyzed the data, S. H., B. W., and G. D. collected the field samples, and S. H. and J. S. led the writing.

## Data Availability

Data for raw site‐by‐explanatory variables and raw taxon‐by‐sites matrices are available from the Dryad Digital Repository: https://doi.org/10.5061/dryad.stqjq2c0g
