# Supplementary material for: Metacommunity Structure of Stream Insects across Three Hierarchical Spatial scales
Source: Ecol Evol. 2020 Mar 3;10(6):2874–84. doi: 10.1002/ece3.6103 (PMC7083666; doi:10.1002/ece3.6103)

**Supporting Information**

Table S1. List of stream insects and their classification to dispersal groups. Drifting propensity was categorized as low (catastrophic only), and high (typically observed or dominant in drift samples). Female dispersal potential was categorized as Low (< 1 km before laying eggs), and high (fly > 1 km before lying eggs).

| Order | Family | Drifting propensity | Female dispersal |
| --- | --- | --- | --- |
| Coleoptera | Dytiscidae | Low | High |
|  | Elmidae | High | Low |
|  | Haliplidae | Low | Low |
|  | Psephenidae | Low | Low |
| Diptera | Athericidae | Low | Low |
|  | Blephariceridae | Low | Low |
|  | Ceratopogonidae | Low | Low |
|  | Chironominae | High | High |
|  | Orthocladiinae | High | High |
|  | Tanypodinae | High | High |
|  | Empididae | Low | Low |
|  | Psychodidae | Low | Low |
|  | Simuliidae | High | Low |
|  | Tipulidae | Low | Low |
| Ephemeroptera | Baetidae | High | Low |
|  | Caenidae | Low | Low |
|  | Ephemerellidae | High | Low |
|  | Ephemeridae | Low | Low |
|  | Heptageniidae | High | Low |
|  | Isonychiidae | High | Low |
|  | Leptophlebiidae | High | Low |
|  | Siphluriscidae | High | Low |
| Hemiptera | Gerridae | Low | Low |
| Megaloptera | Corydalidae | Low | Low |
| Odonata | Aeshnidae | Low | High |
|  | Cordulegasteridae | Low | High |
|  | Calopterygidae | Low | Low |
|  | Gomphidae | Low | Low |
| Plecoptera | Chloroperlidae | High | Low |
|  | Leuctridae | High | Low |
|  | Nemouridae | High | Low |
|  | Perlidae | Low | High |
|  | Perlodidae | Low | Low |
| Trichoptera | Brachycentridae | Low | Low |
|  | Glossosomatidae | Low | Low |
|  | Goeridae | Low | Low |
|  | Hydropsychidae | High | High |
|  | Hydroptilidae | Low | High |
|  | Lepidostomatidae | Low | Low |
|  | Leptoceridae | Low | High |
|  | Limnephilidae | Low | Low |
|  | Philptamidae | Low | Low |
|  | Psychomyiidae | Low | High |
|  | Polycentropodidae | Low | High |
|  | Rhyacophilidae | Low | Low |

Table S2. The frequency of environmental and spatial variables, connectivity criterion and coefficient α selected in the randomized analyses for all insects at the within-stream (WS) scale, among-stream (AS) scale and among-sub-basin (ASB) scale. Random analysis was performed for all insects with 20, 30, 40 and 50 number of taxa.

|  | *S* = 20 | | | *S* = 30 | | | *S* = 40 | | | *S* = 50 | | |
| --- | --- | --- | --- | --- | --- | --- | --- | --- | --- | --- | --- | --- |
|  | WS | AS | ASB | WS | AS | ASB | WS | AS | ASB | WS | AS | ASB |
| **Environmental variable** |  |  |  |  |  |  |  |  |  |  |  |  |
| Water temperature | 251 | 55 | 325 | 295 | 38 | 354 | 331 | 29 | 380 | 300 | 27 | 337 |
| pH | 72 | 101 | 204 | 34 | 81 | 174 | 4 | 68 | 122 | 33 | 78 | 168 |
| Dissolved oxygen | 308 | 269 | 144 | 387 | 302 | 119 | 450 | **334** | 85 | 384 | 274 | 99 |
| Total dissolved solids | 184 | 286 | 165 | 205 | **321** | 168 | 197 | 329 | 192 | 222 | **323** | 180 |
| Conductivity | 238 | **287** | 144 | 306 | 283 | 125 | 322 | 306 | 129 | 278 | 306 | 151 |
| Calcium concentrations | 162 | 186 | 135 | 0 | 0 | 0 | 0 | 0 | 0 | 0 | 0 | 0 |
| Total nitrogen | 104 | 233 | 170 | 56 | 240 | 184 | 27 | 231 | 180 | 61 | 230 | 183 |
| Total phosphorus | 156 | 191 | 280 | 124 | 212 | 290 | 70 | 239 | 288 | 108 | 215 | 297 |
| Elevation | 186 | 265 | **361** | 146 | 226 | **389** | 119 | 214 | **432** | 145 | 252 | **388** |
| Water depth (cm) | **339** | 127 | 72 | **390** | 94 | 71 | **453** | 62 | 51 | **389** | 93 | 64 |
| **Spatial variable** |  |  |  |  |  |  |  |  |  |  |  |  |
| MEM1 | **301** | 139 | **481** | 224 | 145 | **482** | 165 | 106 | **490** | 233 | 129 | **487** |
| MEM2 | 271 | 207 | 412 | **289** | 184 | 439 | 261 | 172 | 461 | **299** | 216 | 438 |
| MEM3 | 72 | 182 | 31 | 99 | 206 | 18 | 90 | 225 | 16 | 98 | 188 | 21 |
| MEM4 | 140 | **293** | 14 | 186 | **344** | 16 | **263** | **413** | 7 | 161 | **342** | 13 |
| MEM5 | 69 | 39 | 15 | 56 | 24 | 5 | 24 | 12 | 3 | 50 | 30 | 9 |
| MEM6 | 39 | 73 | 16 | 0 | 0 | 0 | 0 | 0 | 0 | 0 | 0 | 0 |
| MEM7 | 106 | 58 | 27 | 82 | 45 | 26 | 74 | 25 | 13 | 94 | 34 | 24 |
| **Connectivity criterion** |  |  |  |  |  |  |  |  |  |  |  |  |
| Delaunay triangulation | 77 | 143 | 81 | 102 | 106 | 106 | 108 | 75 | 157 | 86 | 103 | 114 |
| Gabriel graph | 104 | 35 | 108 | 73 | 32 | 110 | 27 | 65 | 98 | 80 | 29 | 110 |
| Relative neighborhood graph | 59 | 104 | 20 | 71 | 71 | 10 | 155 | 37 | 4 | 69 | 82 | 11 |
| Minimum spanning tree | 0 | 48 | 0 | 0 | 23 | 0 | 0 | 11 | 0 | 0 | 11 | 0 |
| Distance-based | **260** | **170** | **291** | **254** | **268** | **274** | **210** | **312** | **241** | **265** | **275** | **265** |
| ***α*** |  |  |  |  |  |  |  |  |  |  |  |  |
| 1 | 82 | 38 | 64 | 83 | 19 | 67 | 68 | 11 | 86 | 86 | 25 | 65 |
| 2 | 59 | 43 | 52 | 51 | 34 | 63 | 30 | 15 | 67 | 47 | 27 | 61 |
| 3 | 9 | 22 | **73** | 2 | 19 | 55 | 0 | 12 | 43 | 3 | 12 | 40 |
| 4 | 41 | 11 | 30 | 26 | 11 | 21 | 4 | 5 | 27 | 27 | 8 | 30 |
| 5 | 6 | 16 | 9 | 1 | 21 | 17 | 0 | 17 | 17 | 0 | 30 | 27 |
| 6 | 3 | 13 | 8 | 0 | 13 | 14 | 0 | 6 | 17 | 1 | 10 | 15 |
| 7 | 0 | 15 | 8 | 0 | 10 | 7 | 0 | 7 | 10 | 0 | 14 | 7 |
| 8 | 0 | 22 | 6 | 0 | 21 | 7 | 0 | 18 | 4 | 0 | 11 | 6 |
| 9 | 1 | 18 | 4 | 0 | 9 | 4 | 0 | 9 | 6 | 0 | 11 | 1 |
| 10 | 68 | 92 | 71 | 67 | 139 | **87** | 57 | 160 | **103** | 54 | 137 | **74** |
| 1/2 | 34 | 19 | 49 | 63 | 10 | 57 | **143** | 5 | 55 | 70 | 7 | 64 |
| 1/3 | 21 | 7 | 31 | 31 | 5 | 26 | 47 | 4 | 20 | 31 | 6 | 22 |
| 1/4 | 13 | 14 | 7 | 14 | 5 | 7 | 33 | 0 | 2 | 14 | 6 | 15 |
| 1/5 | 8 | 10 | 5 | 8 | 2 | 10 | 18 | 0 | 10 | 7 | 6 | 4 |
| 1/6 | 8 | 4 | 6 | 9 | 1 | 5 | 5 | 2 | 0 | 3 | 2 | 2 |
| 1/7 | 1 | 1 | 2 | 5 | 0 | 3 | 3 | 0 | 0 | 5 | 1 | 1 |
| 1/8 | 6 | 8 | 2 | 6 | 0 | 3 | 0 | 0 | 2 | 3 | 1 | 4 |
| 1/9 | 8 | 1 | 1 | 1 | 1 | 2 | 0 | 1 | 3 | 2 | 0 | 3 |
| 1/10 | **132** | **146** | 72 | **133** | **180** | 45 | 92 | **228** | 28 | **147** | **186** | 59 |

Table S3. The frequency of environmental and spatial variables, connectivity criterion and coefficient α selected in the randomized analyses at the within-stream (WS) scale, among-stream (AS) scale and among-sub-basin (ASB) scale. Random analysis was performed for Low DP (Low drifting propensity), High DP (High drifting propensity), Low FD (Low female dispersal) and High FD (High female dispersal). The highest frequency is shown by bold font.

|  | Low DP | | | High DP | | | Low FD | | | High FD | | |
| --- | --- | --- | --- | --- | --- | --- | --- | --- | --- | --- | --- | --- |
|  | WS | AS | ASB | WS | AS | ASB | WS | AS | ASB | WS | AS | ASB |
| **Environmental variable** |  |  |  |  |  |  |  |  |  |  |  |  |
| Water temperature | 191 | 120 | 305 | 346 | 10 | 357 | 68 | 86 | 219 | **431** | 3 | 470 |
| pH | 127 | 111 | 219 | 1 | 76 | 153 | 372 | 145 | 160 | 0 | 29 | 259 |
| Dissolved oxygen | 58 | 313 | 129 | 477 | 334 | 143 | 72 | **296** | 158 | 320 | **437** | 137 |
| Total dissolved solids | 0 | 296 | 105 | 330 | 278 | 107 | 207 | 154 | 151 | 1 | 384 | 19 |
| Conductivity | 340 | **407** | 296 | 150 | 121 | 68 | 150 | 327 | 186 | 68 | 289 | 17 |
| Calcium concentrations | 35 | 78 | 118 | 55 | 299 | 132 | 205 | 0 | 0 | 281 | 0 | 0 |
| Total nitrogen | 278 | 177 | 44 | 75 | 247 | 204 | 48 | 123 | 174 | 4 | 347 | 298 |
| Total phosphorus | 166 | 28 | **427** | 33 | **351** | 248 | 38 | 196 | 372 | 386 | 273 | 39 |
| Elevation | 336 | 358 | 347 | 78 | 221 | **434** | 362 | 285 | **374** | 86 | 99 | **477** |
| Water depth (cm) | **469** | 112 | 10 | **455** | 65 | 154 | **478** | 150 | 58 | 423 | 63 | 235 |
| **Spatial variable** |  |  |  |  |  |  |  |  |  |  |  |  |
| MEM1 | 315 | 135 | **433** | 179 | 119 | **485** | 326 | 130 | **469** | **496** | 217 | **498** |
| MEM2 | **358** | 143 | 347 | **242** | 128 | 460 | **431** | **341** | 428 | 73 | 43 | 492 |
| MEM3 | 29 | 138 | 140 | 28 | 273 | 16 | 135 | 142 | 32 | 15 | **359** | 1 |
| MEM4 | 213 | **255** | 38 | 217 | **400** | 8 | 35 | 236 | 24 | 40 | 219 | 1 |
| MEM5 | 29 | 178 | 16 | 73 | 13 | 5 | 22 | 13 | 6 | 36 | 38 | 5 |
| MEM6 | 0 | 0 | 0 | 0 | 0 | 0 | 0 | 0 | 0 | 0 | 0 | 0 |
| MEM7 | 17 | 115 | 11 | 135 | 25 | 9 | 16 | 105 | 16 | 38 | 3 | 0 |
| **Connectivity criterion** |  |  |  |  |  |  |  |  |  |  |  |  |
| Delaunay triangulation | 25 | 11 | 64 | 144 | 87 | 132 | 18 | 145 | 120 | 9 | 10 | 7 |
| Gabriel graph | 9 | **225** | 157 | 105 | 23 | 57 | 12 | 51 | 100 | **381** | 103 | 4 |
| Relative neighborhood graph | 48 | 107 | 82 | 71 | 52 | 8 | 24 | 80 | 49 | 31 | 72 | 0 |
| Minimum spanning tree | 0 | 77 | 0 | 0 | 9 | 0 | 0 | 57 | 0 | 0 | 4 | 0 |
| Distance-based | **418** | 80 | **197** | **180** | **329** | **303** | **446** | **167** | **231** | 79 | **311** | **489** |
| ***α*** |  |  |  |  |  |  |  |  |  |  |  |  |
| 1 | 2 | 36 | 58 | 45 | 14 | 60 | **292** | 41 | 38 | 29 | 37 | 11 |
| 2 | 17 | 25 | 58 | 103 | 11 | 75 | 32 | 47 | 67 | 64 | 22 | 11 |
| 3 | 2 | 17 | 75 | 0 | 6 | 39 | 1 | 43 | 36 | 112 | 11 | 45 |
| 4 | 1 | 15 | 19 | 17 | 4 | 26 | 0 | 20 | 19 | **247** | 1 | 50 |
| 5 | 0 | 19 | 17 | 4 | 9 | 21 | 1 | 20 | 10 | 10 | 9 | 9 |
| 6 | 5 | 12 | 16 | 0 | 8 | 11 | 0 | 56 | 14 | 4 | 7 | 1 |
| 7 | 7 | 20 | 9 | 0 | 6 | 6 | 2 | 30 | 13 | 2 | 6 | 5 |
| 8 | 9 | 14 | 4 | 0 | 15 | 6 | 0 | 25 | 6 | 0 | 0 | 2 |
| 9 | 9 | 9 | 6 | 0 | 9 | 6 | 0 | 19 | 13 | 0 | 0 | 4 |
| 10 | 41 | 95 | **153** | 9 | **212** | **94** | 11 | 35 | 73 | 0 | 22 | **360** |
| 1/2 | 5 | 39 | 30 | 76 | 2 | 58 | 19 | 23 | 38 | 2 | 43 | 0 |
| 1/3 | 16 | 3 | 8 | 90 | 2 | 21 | 20 | 20 | 15 | 0 | 6 | 0 |
| 1/4 | 3 | 3 | 6 | 20 | 2 | 6 | 7 | 3 | 10 | 0 | 1 | 1 |
| 1/5 | 11 | 9 | 5 | 4 | 1 | 7 | 6 | 5 | 5 | 0 | 0 | 1 |
| 1/6 | 8 | 6 | 5 | 2 | 3 | 2 | 8 | 2 | 4 | 4 | 0 | 0 |
| 1/7 | 3 | 0 | 2 | 3 | 0 | 3 | 7 | 5 | 4 | 3 | 1 | 0 |
| 1/8 | 0 | 21 | 3 | 0 | 0 | 1 | 4 | 10 | 1 | 0 | 1 | 0 |
| 1/9 | 1 | 0 | 0 | 1 | 0 | 2 | 3 | 1 | 4 | 1 | 0 | 0 |
| 1/10 | **360** | **157** | 26 | **126** | 196 | 56 | 87 | **95** | **130** | 22 | **333** | 0 |

Table S4. Mean and standard deviation (SD) of variance components from the Classical and MSR-based variance partitioning (VP) for randomly constructed insects at the within-stream scale, among-stream scale and among-sub-basin scale. *S*: the number of randomized taxa. Adj. R^2^: adjusted coefficient of determination; [E|S]: purely environmental variation; [E∩S]: variation from correlations between environmental and spatial variables; [S|E]: purely spatial variation.

| Taxa group | *S* | Spatial scale | VP Type | Adj. R^2^ | [E\|S] | [E∩S] | [S\|E] |
| --- | --- | --- | --- | --- | --- | --- | --- |
| All insect | 20 | within-stream | Classical | Mean | 0.165 | 0.047 | 0.114 |
|  |  |  |  | SD | 0.105 | 0.057 | 0.111 |
|  |  |  | MSR-based | Mean | 0.108 | 0.058 | 0.100 |
|  |  |  |  | SD | 0.076 | 0.047 | 0.120 |
|  | 20 | among-stream | Classical | Mean | 0.101 | 0.107 | 0.065 |
|  |  |  |  | SD | 0.099 | 0.069 | 0.068 |
|  |  |  | MSR-based | Mean | 0.054 | 0.110 | 0.067 |
|  |  |  |  | SD | 0.050 | 0.052 | 0.075 |
|  | 20 | among-sub-basin | Classical | Mean | 0.108 | 0.222 | 0.067 |
|  |  |  |  | SD | 0.067 | 0.096 | 0.062 |
|  |  |  | MSR-based | Mean | 0.059 | 0.162 | 0.126 |
|  |  |  |  | SD | 0.039 | 0.062 | 0.093 |
| All insect | 30 | within-stream | Classical | Mean | 0.178 | 0.032 | 0.109 |
|  |  |  |  | SD | 0.089 | 0.046 | 0.085 |
|  |  |  | MSR-based | Mean | 0.117 | 0.042 | 0.084 |
|  |  |  |  | SD | 0.064 | 0.037 | 0.090 |
|  | 30 | among-stream | Classical | Mean | 0.099 | 0.092 | 0.054 |
|  |  |  |  | SD | 0.093 | 0.056 | 0.055 |
|  |  |  | MSR-based | Mean | 0.048 | 0.102 | 0.052 |
|  |  |  |  | SD | 0.044 | 0.042 | 0.062 |
|  | 30 | among-sub-basin | Classical | Mean | 0.094 | 0.215 | 0.058 |
|  |  |  |  | SD | 0.054 | 0.076 | 0.048 |
|  |  |  | MSR-based | Mean | 0.053 | 0.148 | 0.123 |
|  |  |  |  | SD | 0.032 | 0.047 | 0.073 |
| All insect | 40 | within-stream | Classical | Mean | 0.193 | 0.016 | 0.118 |
|  |  |  |  | SD | 0.067 | 0.030 | 0.065 |
|  |  |  | MSR-based | Mean | 0.126 | 0.028 | 0.079 |
|  |  |  |  | SD | 0.046 | 0.025 | 0.062 |
|  | 40 | among-stream | Classical | Mean | 0.112 | 0.081 | 0.058 |
|  |  |  |  | SD | 0.089 | 0.051 | 0.050 |
|  |  |  | MSR-based | Mean | 0.050 | 0.095 | 0.053 |
|  |  |  |  | SD | 0.040 | 0.036 | 0.057 |
|  | 40 | among-sub-basin | Classical | Mean | 0.088 | 0.219 | 0.058 |
|  |  |  |  | SD | 0.045 | 0.063 | 0.043 |
|  |  |  | MSR-based | Mean | 0.051 | 0.142 | 0.134 |
|  |  |  |  | SD | 0.028 | 0.038 | 0.063 |
| All insect | 50 | within-stream | Classical | Mean | 0.174 | 0.032 | 0.112 |
|  |  |  |  | SD | 0.084 | 0.045 | 0.112 |
|  |  |  | MSR-based | Mean | 0.114 | 0.047 | 0.087 |
|  |  |  |  | SD | 0.059 | 0.040 | 0.121 |
|  | 50 | among-stream | Classical | Mean | 0.101 | 0.093 | 0.062 |
|  |  |  |  | SD | 0.095 | 0.059 | 0.057 |
|  |  |  | MSR-based | Mean | 0.050 | 0.100 | 0.061 |
|  |  |  |  | SD | 0.045 | 0.043 | 0.062 |
|  | 50 | among-sub-basin | Classical | Mean | 0.097 | 0.219 | 0.062 |
|  |  |  |  | SD | 0.057 | 0.081 | 0.051 |
|  |  |  | MSR-based | Mean | 0.054 | 0.149 | 0.129 |
|  |  |  |  | SD | 0.034 | 0.049 | 0.076 |
| Low drifting propensity | 20 | within-stream | Classical | Mean | 0.085 | 0.030 | 0.060 |
|  |  |  |  | SD | 0.048 | 0.034 | 0.054 |
|  |  |  | MSR-based | Mean | 0.049 | 0.034 | 0.045 |
|  |  |  |  | SD | 0.031 | 0.029 | 0.044 |
|  | 20 | among-stream | Classical | Mean | 0.165 | 0.090 | 0.042 |
|  |  |  |  | SD | 0.084 | 0.072 | 0.040 |
|  |  |  | MSR-based | Mean | 0.089 | 0.123 | 0.025 |
|  |  |  |  | SD | 0.042 | 0.060 | 0.037 |
|  | 20 | among-sub-basin | Classical | Mean | 0.114 | 0.154 | 0.085 |
|  |  |  |  | SD | 0.049 | 0.058 | 0.063 |
|  |  |  | MSR-based | Mean | 0.065 | 0.119 | 0.119 |
|  |  |  |  | SD | 0.031 | 0.044 | 0.066 |
| High drifting propensity | 20 | within-stream | Classical | Mean | 0.215 | 0.012 | 0.133 |
|  |  |  |  | SD | 0.081 | 0.024 | 0.079 |
|  |  |  | MSR-based | Mean | 0.146 | 0.029 | 0.085 |
|  |  |  |  | SD | 0.060 | 0.027 | 0.090 |
|  | 20 | among-stream | Classical | Mean | 0.110 | 0.080 | 0.070 |
|  |  |  |  | SD | 0.095 | 0.056 | 0.058 |
|  |  |  | MSR-based | Mean | 0.052 | 0.091 | 0.066 |
|  |  |  |  | SD | 0.042 | 0.037 | 0.065 |
|  | 20 | among-sub-basin | Classical | Mean | 0.096 | 0.245 | 0.051 |
|  |  |  |  | SD | 0.059 | 0.082 | 0.045 |
|  |  |  | MSR-based | Mean | 0.054 | 0.171 | 0.123 |
|  |  |  |  | SD | 0.034 | 0.048 | 0.069 |
| Low female dispersal | 20 | within-stream | Classical | Mean | 0.169 | 0.028 | 0.030 |
|  |  |  |  | SD | 0.059 | 0.039 | 0.041 |
|  |  |  | MSR-based | Mean | 0.113 | 0.062 | 0.015 |
|  |  |  |  | SD | 0.048 | 0.040 | 0.036 |
|  | 20 | among-stream | Classical | Mean | 0.173 | 0.078 | 0.066 |
|  |  |  |  | SD | 0.084 | 0.058 | 0.060 |
|  |  |  | MSR-based | Mean | 0.099 | 0.113 | 0.039 |
|  |  |  |  | SD | 0.046 | 0.050 | 0.051 |
|  | 20 | among-sub-basin | Classical | Mean | 0.159 | 0.167 | 0.037 |
|  |  |  |  | SD | 0.068 | 0.071 | 0.045 |
|  |  |  | MSR-based | Mean | 0.086 | 0.157 | 0.050 |
|  |  |  |  | SD | 0.040 | 0.055 | 0.055 |
| High female dispersal | 15 | within-stream | Classical | Mean | 0.059 | 0.048 | 0.278 |
|  |  |  |  | SD | 0.056 | 0.057 | 0.135 |
|  |  |  | MSR-based | Mean | 0.023 | 0.035 | 0.289 |
|  |  |  |  | SD | 0.029 | 0.043 | 0.136 |
|  | 20 | among-stream | Classical | Mean | 0.155 | 0.053 | 0.078 |
|  |  |  |  | SD | 0.108 | 0.041 | 0.062 |
|  |  |  | MSR-based | Mean | 0.064 | 0.092 | 0.053 |
|  |  |  |  | SD | 0.042 | 0.041 | 0.068 |
|  | 20 | among-sub-basin | Classical | Mean | 0.044 | 0.310 | 0.026 |
|  |  |  |  | SD | 0.035 | 0.064 | 0.032 |
|  |  |  | MSR-based | Mean | 0.026 | 0.201 | 0.127 |
|  |  |  |  | SD | 0.022 | 0.041 | 0.058 |

Figure S1. Map of high female dispersal abundance in 18 sites at the within-stream scale.


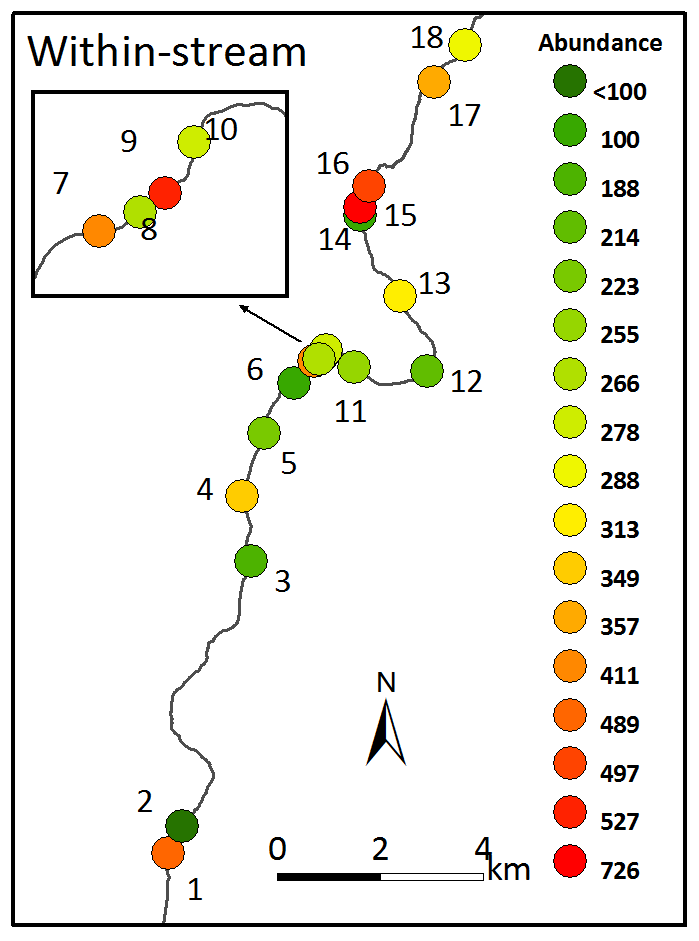

Supplement: Supplementary file 1 [file ECE3-10-2874-s001.docx]
